# Comparative Analysis of the Liver Transcriptome among Cattle Breeds Using RNA-seq

**DOI:** 10.3390/vetsci6020036

**Published:** 2019-03-29

**Authors:** Chandra Shekhar Pareek, Mateusz Sachajko, Jedrzej M. Jaskowski, Magdalena Herudzinska, Mariusz Skowronski, Krzysztof Domagalski, Joanna Szczepanek, Urszula Czarnik, Przymeslaw Sobiech, Dominika Wysocka, Mariusz Pierzchala, Ewa Polawska, Kamila Stepanow, Magdalena Ogłuszka, Edyta Juszczuk-Kubiak, Yaping Feng, Dibyendu Kumar

**Affiliations:** 1 Centre for Modern Interdisciplinary Technologies, Nicolaus Copernicus University, 87-100 Torun, Poland; mateuszsachajko@doktorant.umk.pl (M.S.); krydom@umk.pl (K.D.); szczepanekj@umk.pl (J.S.); 2 Centre of Veterinary Sciences, Nicolaus Copernicus University, 87-100 Torun, Poland; jmjaskowski@umk.pl (J.M.J.); mherudzinska@umk.pl (M.H.); skowron@umk.pl (M.S.); 3 Faculty of Animal Bio-engineering, University of Warmia and Mazury in Olsztyn, 10-719 Olsztyn, Poland; czar@uwm.edu.pl; 4 Faculty of Veterinary Medicine, University of Warmia and Mazury in Olsztyn, 10719 Olsztyn, Poland; psobiech@uwm.edu.pl (P.S.); dominika.wysocka@uwm.edu.pl (D.W.); 5 Institute of Genetics and Animal Breeding of the Polish Academy of Sciences, 05-552 Jastrzębiec, Poland; m.pierzchala@ighz.pl (M.P.); ewchubby@poczta.onet.pl (E.P.); k.stepanow@ighz.pl (K.S.); m.ogluszka@ighz.pl (M.O.); 6 Faculty of Veterinary Medicine, Warsaw University of Life Sciences, 02-787 Warsaw, Poland; ekubiak2@gmail.com; 7 Waksman Institute of Microbiology, Rutgers, The State University of New Jersey, Piscataway, NJ 08 854, USA; ypfeng@waksman.rutgers.edu (Y.F.); dk@waksman.rutgers.edu (D.K.)

**Keywords:** liver, cattle, transcriptome, gene expression, gene-transcripts, GO terms, pathway enrichment, cytoscape-Clue-GO, Polish-Red, Polish-HF, Hereford, RNA-seq

## Abstract

Global gene expression in liver transcriptome varies among cattle breeds. The present investigation was aimed to identify the differentially expressed genes (DEGs), metabolic gene networks and metabolic pathways in bovine liver transcriptome of young bulls. In this study, we comparatively analyzed the bovine liver transcriptome of dairy (Polish Holstein Friesian (HF); *n* = 6), beef (Hereford; *n* = 6), and dual purpose (Polish-Red; *n* = 6) cattle breeds. This study identified 895, 338, and 571 significant (*p* < 0.01) differentially expressed (DE) gene-transcripts represented as 745, 265, and 498 hepatic DE genes through the Polish-Red versus Hereford, Polish-HF versus Hereford, and Polish-HF versus Polish-Red breeds comparisons, respectively. By combining all breeds comparisons, 75 hepatic DE genes (*p* < 0.01) were identified as commonly shared among all the three breed comparisons; 70, 160, and 38 hepatic DE genes were commonly shared between the following comparisons: (i) Polish-Red versus Hereford and Polish-HF versus Hereford; (ii) Polish-Red versus Hereford and Polish-HF versus Polish-Red; and (iii) Polish-HF versus Hereford and Polish-HF versus Polish-Red, respectively. A total of 440, 82, and 225 hepatic DE genes were uniquely observed for the Polish-Red versus Hereford, Polish-HF versus Hereford, and Polish-Red versus Polish-HF comparisons, respectively. Gene ontology (GO) analysis identified top-ranked enriched GO terms (*p* < 0.01) including 17, 16, and 31 functional groups and 151, 61, and 140 gene functions that were DE in all three breed liver transcriptome comparisons. Gene network analysis identified several potential metabolic pathways involved in glutamine family amino-acid, triglyceride synthesis, gluconeogenesis, p38MAPK cascade regulation, cholesterol biosynthesis (Polish-Red versus Hereford); IGF-receptor signaling, catecholamine transport, lipoprotein lipase, tyrosine kinase binding receptor (Polish-HF versus Hereford), and PGF-receptor binding, (Polish-HF versus Polish-Red). Validation results showed that the relative expression values were consistent to those obtained by RNA-seq, and significantly correlated between the quantitative reverse transcription PCR (RT-qPCR) and RNA-seq (Pearson’s r  > 0.90). Our results provide new insights on bovine liver gene expressions among dairy versus dual versus beef breeds by identifying the large numbers of DEGs markers submitted to NCBI gene expression omnibus (GEO) accession number GSE114233, which can serve as useful genetic tools to develop the gene assays for trait-associated studies as well as, to effectively implement in genomics selection (GS) cattle breeding programs in Poland.

## 1. Introduction

With the advancement of next-generation genome sequencing (NGS) technology, transcriptome complexity and dynamics can now be explored at different levels [1]. RNA-sequencing (RNA-seq) has revolutionized sequencing-based technology [2], and is commonly used for characterizing and comparing the gene expression profiling to identify genome-wide differentially expressed (DE) gene-transcripts between two or more biological conditions of interest in various tissue samples. A comparison of transcriptome of taurine (*Bos taurus*) breeds provides not only a high-resolution survey of the gene expression variation at different levels, but also provides important biological insights into the phenotypic differentiation among cattle breeds [3]. In Poland, the Polish-Red and Polish Holstein-Friesian (Polish-HF) are the two native taurine breed reared in active breeding population [4]. The Polish-HF is characterized as dairy breed, while Polish-Red is characterized as dual purpose [5]. Both native breeds are used for the beef purpose in Poland, as in many countries of Central Europe [4,6]. In recent studies, it has been well documented that gene expression of different bovine transcriptome, such as *Longissimus thoracis* [7], blastocysts [8,9], liver [10,11,12,13,14,15], mammary gland [16], adipose tissue [17], horn cancer [18], milk fat globule [19], and subcutaneous adipose tissue [20], varies among cattle breeds. However, little has been elucidated regarding the expression of genes and its regulation mechanism in global cattle breeds, including the native Polish cattle breeds. Thus, understanding the transcriptomic variation among global cattle breeds is an essential pre-requisite to unveil the mechanistic knowledge on their differentiation on phenotypes, including appearance, physiological, behavioral, and production traits [3]. Most of the recent RNA-seq studies that have been performed in cattle were focused mainly on identifying the differentially expressed gene (DEG) variations that influence economically important traits in cattle [21,22,23,24,25,26]. This genetic variation information based on DEG markers has increasingly been used in cattle breeding improvement programs through gene-assisted selection (GAS) and genomic selection (GS), to improve conventional phenotype selection. In general, the identification of DEG markers for particular economically important traits has great potential for the genetic improvement of cattle, and the implementation of GAS and GS programs has been highly recommended for use in cattle breeding programs worldwide [27,28]. The liver transcriptome is central to most of the economically important metabolic processes in cattle, and has a major influence on the genetic improvement of production trait variation, namely, feeding efficiencies in dairy and beef breeds [10,11,12,13,14], carcass trait including muscle growth and development in young growing cattle breeds [5,7,24,25,26]. In this study, we aimed to compare the global gene expression across the entire liver transcriptome in young growing bulls aged between 6 to 12 months using RNA-seq to identify the DE gene-transcripts in bovine liver among cattle breeds (Polish-Red for dual purpose, Polish-HF for dairy, and Hereford for beef), to compare the DE-gene transcripts among dairy versus dual versus beef breeds, and to improve the liver transcriptome annotation of the bovine genome.

## 2. Materials and Methods

### 2.1. Animals and Biological Sample Collection

A total of 18 young bulls aged between 6 to 12 months representing Polish-HF, Polish-Red and Hereford cattle breeds (*n* = 6, for each breed) were investigated using RNA-seq. In this paper, we only investigated the comparison of liver transcriptome among three cattle breeds. The age groups within the cattle breed were not analyzed and investigated because of small sample size. However, a total of 44 young bulls were investigated to validate the RNA-seq experiment using qRT-PCR. The investigated animals were purchased at birth randomly. Just after birth, they were brought to the experimental herd/farm of Institute of Genetics and Animal Breeding (IGAB), Polish Academy of Science (PAS), Jastrzębiec, Poland and reared under uniform environment and feeding system. After the systematically slaughtering of young bulls, the collected liver tissues samples were immediately kept in liquid nitrogen and finally stored in a deep freezer at –80 °C. All procedures involving rearing of animals were performed in accordance with the guiding principles for the care and use of research animals, and were approved by the local ethics commission (permission No. 3/2005) of IGAB, PAS, Jastrzębiec, Poland. The phenotypic database of investigated animals was summarized in Table 1.

### 2.2. Laboratory Methods

Complete workflow of RNA-seq laboratory method is presented in Figure S1. Total RNA was extracted and prepared from 50–60 mg of frozen bovine liver tissues (*n* = 18) using the guanidinium thiocyanate method [29] (TRIzol reagent: Thermo Fisher Scientific Inc., Waltham, MA, USA). The total RNA from each sample was further purified to remove the genomic DNA contamination using the RNase-free DNase clean-up kit (Thermo Fisher Scientific Inc., Waltham, MA, USA). The RNA quality and quantity were assessed using automated capillary gel electrophoresis on a Bioanalyzer 2100 with RNA 6000 Nano Labchips according to manufacturer’s instructions (Agilent Technologies, Palo Alto, CA, USA). The RNA integrity number (RIN) values of all the biological samples (*n* = 18) ranged from 6.9 to 8.5 (Figures S2–S4). A total 5 μg total RNA were used for mRNA isolation, and two biological replicates were used for each biological sample. The dUTP directional mRNA libraries preparation were carried out using the Dynabeads mRNA Direct™ kit (Thermo Fisher Scientific Inc., Waltham, MA, USA), and NEBNext Ultra Directional RNA library preparation Kit for Illumina according to manufacturer’s instructions (New England Bio Labs, Hitchin, UK). The cDNA fragments were end-repaired, A-tailed, and ligated to the TruSeq y-tail single indexes using Illumina TruSeq DNA kit, followed by cutting of the indexed libraries with user enzyme, and PCR amplifications for 12 cycles. Finally, to get the highest quality data on NextSeq 500 Illumina sequencing platform, optimum cluster deposition was made by quantitation of libraries using qPCR according to the Illumina sequencing library qPCR quantification guide (Kapa Biosystems, Wilmington, MA, USA). Finally, 156 × 156 bp paired-end sequence reads were generated using the Illumina NextSeq 500 platform high output/300 cycle kits [30,31].

### 2.3. Sequence Quality Control and Read Mapping

Adaptors were removed using Cutadapt software [32]. The minimum overlap length was set to 10 and the error rate was set to 0.05. After cutting the adaptor, the low-quality bases were trimmed with an average score of 15 for five consecutive bases from the 3’-end. The processed reads were mapped to the Ensembl75_UMD3-1.1 reference genome (https://oct2018.archive.ensembl.org/Bos_taurus/Info/Index) based the Hereford cow, L1 Dominette 01449 (http://bovinegenome.elsiklab.missouri.edu/Apollo2/22875/jbrowse/index.html) and to the Y chromosome from the Btau_4.6.1 assembly using Bowtie2 through TopHat [33], and the HTSeq framework version 0.5.3p9 [34] used to count the number of aligned reads for each gene. The current reference Bos taurus genome assembly is represented by 19,994 and 26,740 annotated coding genes and gene transcripts (https://oct2018.archive.ensembl.org/Bos_taurus/Info/Annotation). The FASTQ sequencing data of this present study were deposited in the NCBI database under submission number: SRS1296732 (http://www.ncbi.nlm.nih.gov/sra?linkname=bioproject_sra_all&from_uid=312148) [31] and the gene expression data of investigated animals (*n* = 18) were deposited in the NCBI database under submission number: GSE 114233 (https://www.ncbi.nlm.nih.gov/geo/query/acc.cgi?acc=GSE114233) and summarized in Table 2.

### 2.4. Breed Comparisons Analysis of RNA-seq Read Count Data Using DEseq and EdgeR Bioconductor Packages

Bioconductor is the most popular and commonly used bioinformatics tools for the analysis of RNA-seq read count data using the R statistical programming language [35]. In our study, we used two R Bioconductor packages (v2.14.1, Open source software for bioinformatics, Boston, MA, USA), the DEseq [36] and EdgeR [37,38], for the normalization of RNA-seq DE gene-transcripts data generated by comparing the liver transcriptomes of Polish-HF, Polish-Red, and Hereford cattle. Although they are similar in terms of differential analysis, they differ in dispersion estimation. DEseq is more conservative [39], while EdgeR is more sensitive to outliers [40]. Prior to DE gene-transcript analysis, the read counts were adjusted for each sequenced library (*n* = 18) using the DEseq and EdgeR packages with one normalized scaling factor.

The DEseq R package 1.12.0 (https://bioconductor.org/packages/release/bioc/html/DESeq.html) was used to analyze DE with the DEseq pipeline approach, and the Benjamini and Hochberg method [41] was used to correct the p-values. The EdgeR GLM approach was applied to determine the DE gene-transcripts between cattle breeds using the trimmed mean of M values (TMM) normalisation method [42]. For both pipelines, the false discovery rate (FDR) adjustments were performed to account for multiple testing in the DE gene-transcript comparisons among the three breeds. The DE gene-transcripts with an adjusted two-sided *p*-value of ≤ 0.01 that showed a greater than 2-fold change in expression was considered differentially expressed. The DEseq and EdgeR platforms were used to perform pairwise comparisons among breeds using parametric tests, where the read-counts followed a negative binomial distribution with a gene-specific dispersion parameter. These packages mainly differ in estimation of the dispersion parameter and the type of normalization they follow. The DEseq and EdgeR programs normalize the read-count per gene based on the total gene depth per individual. These two methods were selected based on evidence in the literature of their robustness. For DEseq, the DE gene-transcripts were defined as those genes with an absolute log fold change (logFC) of > 1 and an adjusted *p*-value of ≤ 0.01 as the threshold, whereas for EdgeR, the DE gene-transcripts with a logFC of > 1 and an adjusted FDR of ≤ 0.01 were adopted as the standard for judging statistically significant differences in gene expression. In our study, three cattle breed comparisons were performed using the DEseq and EdgeR packages, and the DE Gene-transcripts were identified by comparing the bovine liver transcriptome between (i) Polish-Red versus Hereford, (ii) Polish-HF versus Hereford, and (iii) Polish-HF versus Polish-Red cattle breeds. After identification of DE gene-transcripts, RNA-seq data normalization by the *p*-value and FDR calculation, the resulting expression intensity values were further visualized based on the heat-map plots and Venn diagrams using the standard protocols [43,44,45,46,47].

### 2.5. Comparative Analysis of GO Terms among Cattle Breeds Using TopGO and ClueGO Packages

To understand the biological differences between cattle breeds based on the differential expression analyses, we carried out GO terms analysis of DE gene-transcripts to annotate the genes to biological/cellular/molecular terms in a hierarchically structured way using TopGO [48] enrichment analysis for biological processes. Furthermore, the functional distribution of GO terms for bovine liver tissues of cattle breeds were analyzed to assign genes to functional pathways, as well as, to interpret and visualize the functionally group terms in the form of gene networks and pathways charts using Cytoscape-ClueGO [49,50]. The ClueGO plug-in can extrapolate the biological function of large gene lists by identifying significant GO terms and Kyoto Encyclopedia of Genes and Genomes (KEGG) pathways [51,52]. It also facilitates the visualization of functionally related genes displayed as a clustered network and chart. The statistical test used for the enrichment was based on a right-sided hypergeometric option with a Benjamini–Hochberg correction for multiple testing (FDR < 0.05) and a kappa score of 0.3. The ClueGO software calculates enrichment scores for selected sets of genes against a user-provided gene list [48,49,50].

### 2.6. Validation of DE Gene-Transcripts Using RT-PCR/qPCR

A total of eight DE gene-transcripts primers set were designed based on the significant DE gene-transcripts identified by RNA-seq experiment. For the Polish-Red versus Hereford comparison: gene encoding calpain 11 (CAPN11), Insulin-like growth factor level (IGF-I), and Bos taurus insulin-like growth factor binding protein, acid labile subunit (IGFALS); for the Polish-HF versus Hereford comparison: gene encoding Calpain-2 catalytic subunit (CAPN2), insulin like growth factor binding protein 2 (IGFBP2), and growth hormone (GH); and for the Polish-HF versus Polish-Red comparison: gene encoding insulin like growth factor binding protein 1 (IGFBP1) and family with sequence similarity 13 member A (FAM13A) DEGs markers were selected (Table 3). The primer pairs were designed between exons to prevent false positive amplification from contaminating genomic DNA.

#### 2.6.1. RT-PCR/qPCR

A total of 44 RNA samples were treated with DNase I (Thermo Fisher Scientific, Cleveland, OH, USA) and reverse-transcribed using a Roche RT-PCR reagent kit (Transcriptor high fidelity cDNA synthesis kit, Roche, Basel, Switzerland) in the presence of random hexamers. The cDNA samples were quantified on a Roche 480 LightCycler^®^ system using the qPCR SYBR Green I Master (Roche). Approximately 1  μg of RNA was used as the template for qPCR using the primer sets listed in Table 3, with cycling conditions of  10 min at 95 °C followed by 40 cycles of 5 s at 95 °C, 15 s at 60 °C, and 20 s at 72 °C. The *glyceraldehyde-3-phosphate dehydrogenase* (*GAPDH*) gene transcript was used as the internal control [53].

#### 2.6.2. qPCR Statistics

The sample cycle threshold (CT) values were standardized for each template using the *GAPDH* reference gene as a control, and a robust qPCR efficiency assessments method [54] was used to analyze the relative change in gene expression from the qPCR experiments. Three biological replicates and three replicate reactions per sample were used to ensure statistical credibility. The expression levels of each of the identified DE gene-transcripts determined by qPCR were analyzed using the Mann-Whitney. Two-tailed *p*-values were used for all analyses, and *p*-values < 0.01 were considered statistically significant. Statistical analyses and production of Pearson’s correlation graphs [55] were performed with IBM SPSS 20 (IBM Corp. Released 2011, IBM SPSS Statistics for Windows, Version 20.0, Armonk, NY, USA) and GraphPad Prism 6 software (GraphPad Software, San Diego, CA, USA).

## 3. Results

### 3.1. Comparison of Liver Transcriptome in Cattle Breeds

Using the DEseq and EdgeR pipelines, the bovine liver transcriptome were compared across three cattle breeds; hepatic non-DE gene-transcripts and hepatic DE genes were identified and compiled into the following datasets: (i) Polish-Red versus Hereford (Table S1), (ii) Polish-HF versus Hereford (Table S2), and (iii) Polish-HF versus Polish-Red (Table S3). All the filtered non-DE gene-transcripts without any cut-off p-values formed the hepatic non-DE gene-transcripts dataset (Tables S1–S3: Sheet-2), whereas all the filtered DE gene-transcripts with an adjusted cut-off *p*-value of < 0.01 formed the dataset of significant hepatic DE genes (Tables S1–S3: sheet-3). By comparing all the filtered non-DE gene-transcripts without any cut-off *p*-values of bovine liver transcriptome, a total of 51,575, 51,560, and 50,985 hepatic gene-transcripts, represented as 18,217, 18,201, and 17,977 hepatic genes (Tables S1–S3: Sheet-2), were identified in the breed comparison of Polish-Red versus Hereford, Polish-HF versus Hereford, and Polish-HF versus Polish-Red cattle, respectively. Similarly, by comparing the bovine liver transcriptome with an adjusted cut-off *p*-value of < 0.01, a total of 895, 338, and 571 significant hepatic DE gene-transcripts, represented as 745, 265, and 498 hepatic DE genes (Tables S1–S3: Sheet-3), were identified in the breed comparison of Polish-Red versus Hereford, Polish-HF versus Hereford, and Polish-HF versus Polish-Red cattle, respectively.

**Figure 1:**
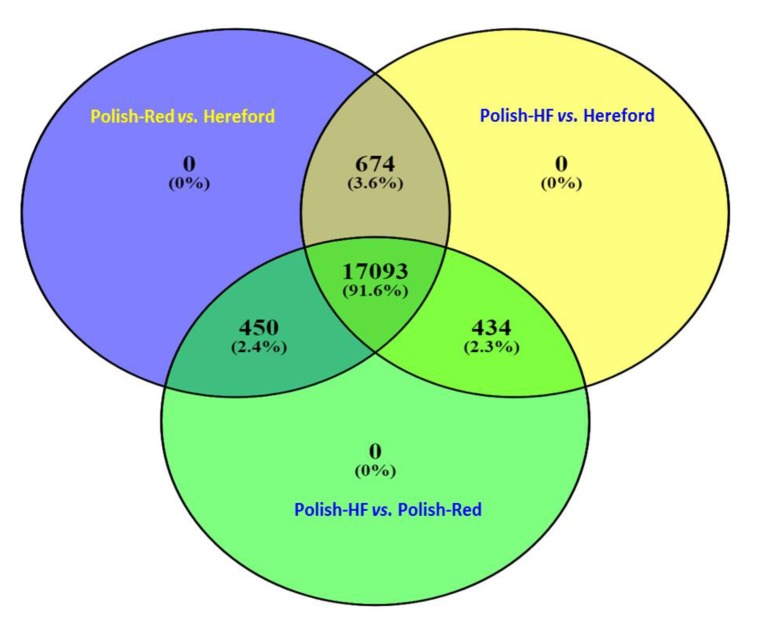
Venn diagram based on the Tables S1–S3 (Excel-Sheet-2) revealed identifications of all the hepatic non-DE genes (without cut-off *p* values) in all three breeds using DEseq and EdgeR pipelines. Figure denotes the comparisons among Polish-Red versus Hereford, Polish-HF versus Hereford, and Polish-HF versus Polish-Red cattle breeds. The numeric values (*n*) of the Venn diagram representing the hepatic non-DE genes are listed in Table S4.

Furthermore, all three breed comparisons were analyzed using online Venn diagram web resources (http://bioinformatics.psb.ugent.be/webtools/Venn/). In case of all the filtered hepatic gene-transcripts without any cut-off p-values, the Venn diagram revealed that the majority of hepatic gene-transcripts (*n* = 17,093) were commonly shared among all three breed comparisons; a total of 674, 450, and 434 hepatic gene-transcripts were commonly shared between the following comparisons: (i) Polish-Red versus Hereford and Polish-HF versus Hereford; (ii) Polish-Red versus Hereford and Polish-HF versus Polish-Red; and (iii) Polish-HF versus Hereford and Polish-HF versus Polish-Red, respectively (Figure 1, Table S4).

**Figure 2:**
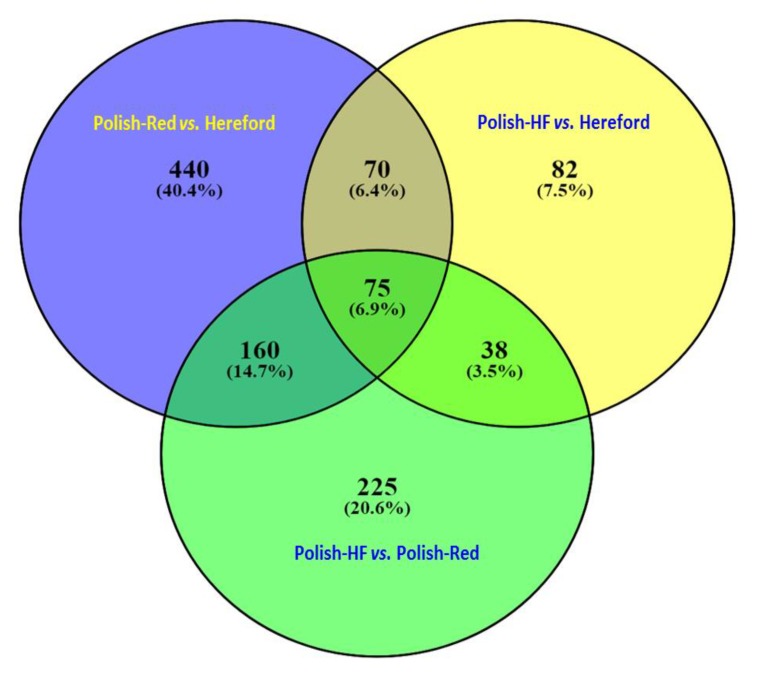
Venn diagram based on the Tables S1–S3 (Excel-Sheet-3) revealed identifications of significant hepatic DE genes (*p* < 0.01) in all three breeds comparisons using DEseq and EdgeR pipelines. Figure denotes the comparisons among Polish-Red versus Hereford, Polish-HF versus Hereford, and Polish-HF versus Polish-Red cattle breeds. The numeric values (*n*) of Venn diagram representing the hepatic DE genes are listed in Table S5.

In case of all the filtered hepatic genes with an adjusted cut-off p-value of < 0.01, the Venn diagram revealed a total of 75 DE hepatic genes that were commonly shared among all three breed comparisons; a total of 70, 160, and 38 DE hepatic genes were commonly shared between the following comparisons: (i) Polish-Red versus Hereford and Polish-HF versus Hereford; (ii) Polish-Red versus Hereford and Polish-HF versus Polish-Red; and (iii) Polish-HF versus Hereford and Polish-HF versus Polish-Red, respectively (Figure 2, Table S5). Moreover, a total of 440, 82, and 225 DE hepatic genes were uniquely observed in the breed comparisons of Polish-Red versus Hereford, Polish-HF versus Hereford, and Polish-Red versus Polish-HF, respectively (Figure 2, Table S5).

### 3.2. Comparison of Gene Ontology Terms among Cattle Breeds Using TopGO Enrichment Analysis

To understand the biological differences between cattle breeds based on the differential expression analyses, we carried out gene ontology (GO) term analysis of DE gene-transcripts to annotate the genes to biological/cellular/molecular terms in a hierarchically structured way using TopGO enrichment analysis for biological processes. The complete list of the top 150 enriched GO terms from all GO-seq analyses (adjusted *p*-value of < 0.01) are summarized in Table S6. For the Polish-Red versus Hereford comparison, the 150 top-ranked GO terms, including the molecular function (MF), biological process (BP), and cellular component (CC) terms, were identified and summarized in Table S6: Sheet 1. For this comparison, the MF GO term, BP GO term, and GO CC term analyses identified some genes overrepresented or most enriched in various biological activities (presented in Table S6: Sheet 1). Similar data are presented in Table S6: Sheet 2 for the Polish-HF versus Hereford comparison, and in Table S6: Sheet 3 for the Polish-HF versus Polish-Red comparison.

### 3.3. Comparison of Gene Ontology Terms among Cattle Breeds Using Cytoscape-ClueGO

#### 3.3.1. Assignment of Genes to Functional Pathways

The functional distribution of GO terms for bovine liver tissues were analyzed to assign the genes to functional pathways using Cytoscape-*ClueGO*. Overall, results revealed a total of 64 gene functional groups and 352 genes with a corresponding function that were assigned to functional pathways in the bovine liver transcriptome in all the three breed comparisons. The corresponding numbers were 17 and 151 for the Polish-Red versus Hereford (Table S7), 16 and 61 for the Polish-HF versus Hereford (Table S8), and 31 and 140 for the Polish-HF versus Polish-Red (Table S9) comparisons.

#### 3.3.2. Interpretation and Visualization of Functionally Group Terms in the Form of Gene Networks and Pathways Charts

Analysis of the biological processes and gene network of all significant DE gene-transcripts (*p* < 0.01) in bovine liver tissue was performed to identify and interpret the GO functionally group terms representing upregulated and downregulated genes (Figures S5–S10), as well as to visualize the functionally grouped networks and pathways with upregulated and downregulated genes (Tables S10–S12), using Cytoscape-*ClueGO*.

The GO terms comparative analysis of Polish-Red versus Hereford identified significantly upregulated GO terms (Figure S5) and gene networks/pathways (Table S10) related to alpha amino acid catabolic process, metabolic processes of triglyceride and unsaturated fatty acid, gluconeogenesis, nucleoside diphosphate phosphorylation, positive regulation of p38MAPK cascade, cholesterol biosynthesis process, and significantly downregulated gene network (Figure S6) and pathways (Table S10) related to regulation of interferon and cytokine production, regulation of actin nucleation, negative regulation of extrinsic apoptotic signaling pathway via death domain receptors, regulation of Iron ion transport and post-embryonic eye morphogenesis.

The GO terms comparative analysis of Polish-HF versus Hereford identified significantly upregulated gene network (Figure S7) and pathways (Table S11) related to IGF receptor signaling, catecholamine transport, quaternary ammonium group transmembrane transporter activity, the regulation of lipoprotein lipase activity and tyrosine kinase binding receptor, and significantly downregulated gene network (Figure S8) and pathways (Table S11) related to positive regulation of glucose import, monocarboxylic acid binding, IGF ternary complex processes, regulation of corticosteroid hormone secretion, negative regulation of tumor necrosis factor production, xenobiotic process, retinoid binding, unsaturated fatty acid metabolic process, steroid metabolic process, and hormone metabolic process.

Finally, the GO terms comparative analysis of Polish-HF versus Polish-Red identified significantly upregulated gene network (Figure S9) and pathways (Table S12) related to negative regulation of viral genome replication, proton symporter activity, post-embryonic eye morphogenesis, platelet-derived growth factor receptor binding, iron ion import into cell and protein activation cascade, and significantly downregulated gene network (Figure S10) and pathways (Table S12) related to cholesterol biosynthetic process, isoprenoid metabolic process, retinoic acid binding, oxidoreductase activity, negative regulation of protein processing, cholesterol efflux and ATP biosynthetic process.

### 3.4. Validation of DE Gene-Transcripts Using Quantitative Real Time PCR (qPCR)

**Figure 3:**
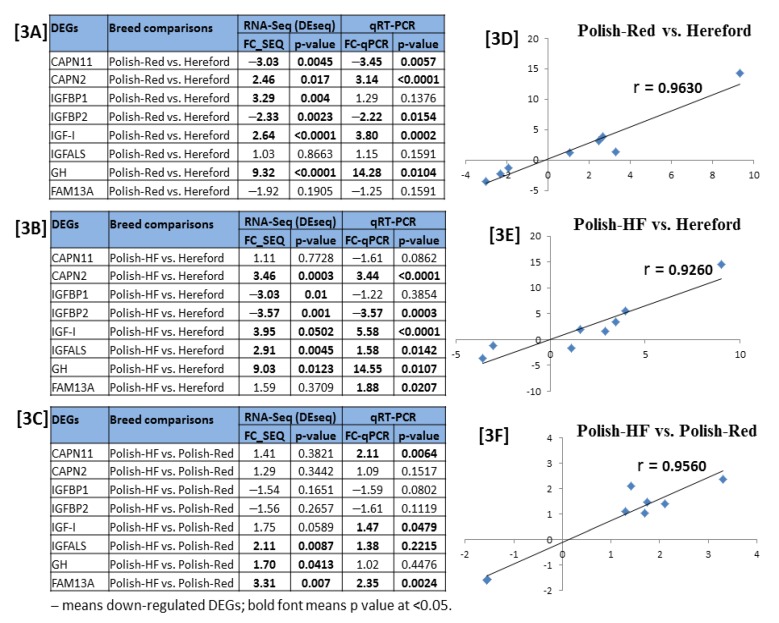
Validation of breed-specific bovine liver RNA-seq experiment via RT-qPCR. (**A**–**C)** denotes the fold change (FC) with *p* values of eight selected DE gene-transcripts calculated with mRNA expressions of breed-specific bovine liver tissues determined via RNA-seq and qPCR, and (**D**–**F)** denotes the correlation of fold changes in gene expression between the RNA-seq and qPCR by Pearson’s correlation analysis.

The eight DE gene-transcripts identified from the RNA-seq experiment were validated by RT-PCR/qPCR assays (Table 3). The fold change values and *p* values of selected DE gene markers represented in both RNA-seq and RT-PCR/qPCR were considered to interpret the DE gene-transcripts validation results (Figure 3A–C). Results revealed that relative gene expression patterns of the selected genes were significantly correlated between the RT-PCR/qPCR and RNA-seq (Pearson’s r  >  0.90; Figure 3D−F). Thus, the RT-PCR/qPCR result largely confirmed the reliability of bovine liver RNA-seq data (Figure 3).

## 4. Discussion

In cattle, the identification of breed-specific DE gene-transcripts is critical findings for the implementation of effective GAS and GS programs to an active global cattle breeding population for genetic improvement of economically important traits [27,28]. In general, the primary goal of genome-wide DE gene-transcripts identification using RNA-seq is to identify significant DE gene-transcripts expressed within the candidate genes (CGs) that provide a complete set of gene expression variations for specific economically important traits. In global cattle breeding practices, the identification of such breed-specific DE gene-transcripts, within the trait-associated CGs can serve as suitable gene assays for trait-associated studies, which can be effectively utilized in GS programs [28].

In our study, large datasets of hepatic gene-transcripts in dairy, dual purpose, and beef cattle breeds (*n* = 154, 121), including significant hepatic DE genes (*n* = 1804), were identified. Moreover, we observed that large numbers (*n* = 170, 93; 91.6%) of identified overlapping hepatic gene-transcripts without any cut-off p-values were shared commonly in all three breeds, in contrast to only 75 (6.9%) significant (*p* < 0.01) DE gene-transcripts. Furthermore, in Figure 1, we did not observed any unique hepatic DE gene-transcripts for the single breed comparison, However, in Figure 2, when the cutoff values were marked (*p* < 0.01), 440 (40.4%), 82 (7.5%), and 225 (20.6%) unique hepatic DE gene-transcripts were observed in dual versus beef, dairy versus beef and dairy versus dual breed comparisons, respectively. From both Figure 1 and Figure 2 data, one can conclude that when hepatic gene transcript data without cutoff values compared, the majority of overlapping unique hepatic gene transcript are sheared in the all three breeds (91.6%) category. In a recent study on comparisons of hepatic expression in Angus, Charolais, and Kinsella Composite (KC) beef breeds similarly identified 96.1% (*n* = 11,636) of the expressed genes that were common to all the three breeds, whereas, a total of 72, 41 and 175 significant DE genes with FDR < 0.01 and Fold change (FC) > 2, were identified in Angus, Charolais, and KC, respectively [13].

Our RNA-seq study reporting for the first time large set gene-expression dataset (https://www.ncbi.nlm.nih.gov/geo/query/acc.cgi?acc=GSE114233) in Polish native cattle breeds. Such hepatic expression dataset for Polish cattle breeds can be very useful to understand the expression profiling of economic trait; for example, Polish Red cattle is characterized by valuable traits such as high resistance to adverse environmental conditions, good health, longevity, good fertility, ease of calving, great calf vigor, and ease of rearing, as well as high biological value of milk. Although the productivity of the breed is poor, the breed may be used for multiple purposes and meets the needs of extensive and ecological breeding [56].

Identification of hepatic DE genes in divergent cattle breeds that are specialized for either milk or meat production or raised as dual-purpose breeds might also have significant impact to detect signatures of selection for economically important dairy and beef production traits [57], as well as detecting potential genomic regions relevant to milk and beef production, which were in good agreement with known quantitative trait loci (QTLs) or candidate genes [58,59]. A recent study on genome-wide SNP analysis of Polish-HF and Polish-Red cattle identified 19 genomic regions encompassing 55 protein-coding genes and numerous quantitative trait loci, which potentially underlined some of the phenotypic traits [60].

In cattle, very few studies have reported the identification of DE genes in the bovine liver transcriptome. In a recent study, the RNA-seq data of liver biopsy samples from 19 dairy cows were used to identify the DEGs between high- and low-feeding efficiency (FE) groups of cows based on residual feed intake (RFI); a comparison between the high and low RFI groups revealed 70 and 19 significant DEGs in Holstein and Jersey cows, respectively [61]. Moreover, a breed comparison study that analyzed the RNA sequence of bovine leukocyte transcriptomes from Holstein, Jersey, and Cholistani breeds identified a total of 165 and 3383 breed-specific DEGs for the Holstein versus Jersey and Holstein versus Cholistani breeds comparisons, respectively. The DEG analysis showed a high similarity between the Holstein and Jersey breeds, and a much greater difference between the taurine and indicine breeds [3]. However, a study by Cesar et al. [62] in Nellore cattle did not identify any DEGs (FDR 10%) in samples with a high linoleic acid or stearic acid content, and very few DEGs for eicosapentaenoic acid (five DEGs), docosahexaenoic acid (four DEGs) and palmitic acid (123 DEGs), while large numbers of DEGs were associated with oleic acid (1134 DEGs) and conjugated linoleic acid (*cis*-9, *trans*-11; 872 DEGs). In another study that evaluated multiple tissues (liver, fat, muscle and pituitary gland) for the sexual dimorphic genes, a total of 24, 14, 86, and 57 tissue-specific DEGs were identified, including gene encoding DEAD (Asp-Glu-Ala-Asp) box polypeptide 3, Y-linked (*DDX3Y*), *ubiquitin specific peptidase 9*, *Y-linked* (*USP9Y*), and zinc finger protein, Y-linked (*ZFY)* that were commonly found in all four tissues [63].

Our identification of breed-specific hepatic DE genes from the three different cattle breeds suggests that the bovine liver transcriptome of different cattle breeds have divergent genetic profiles; for example, the breed-specific shared hepatic DE genes were highest in the Polish-Red versus Hereford and Polish-HF versus Hereford breed comparisons, in contrast to the comparison of the native Polish-HF versus Polish-Red breeds (Figure 1 and Figure 2). This suggests that native (Polish) breeds have a greater number of similar genes in common, or fewer hepatic DE genes, when compared to the Hereford beef breed. This finding could be suggested that the selected dairy and dual-purpose Polish cattle are the typical early maturing breeds that reach puberty at about 264 days old, compared to the Hereford, the typical late-maturing breed that reaches puberty at day 326 [64,65].

Furthermore, by comparing the young bulls from dairy versus dual versus beef breeds hepatic DE genes, our study identified several gene networks and pathways charts of bovine liver active in young growing bulls. When we compared the young bulls from dual versus beef breed: (i) the significant upregulated metabolic pathways for carbohydrate metabolism (gluconeogenesis), fat metabolism (metabolic processes of triglyceride and unsaturated fatty acid, cholesterol biosynthesis process), protein metabolism (alpha amino acid catabolic process), nucleotide metabolism (nucleoside diphosphate phosphorylation), and postnatal muscle growth (positive regulation of p38MAPK cascade); (ii) the highly significant downregulated metabolic pathways for muscle and body growth (regulation of actin nucleation), and molecule transportation and signaling (regulation of Iron ion transport, negative regulation of extrinsic apoptotic signaling pathway via death domain receptors) were identified. However, when we compared the young bulls from dairy versus beef breed, the significant upregulated metabolic pathways for body growth (IGF receptor signalling, catecholamine transport, quaternary ammonium group transmembrane transporter activity), lipid metabolism (the regulation of lipoprotein lipase activity), and energy metabolism (tyrosine kinase binding receptor) were identified; (iii) the significant downregulated metabolic pathways for carbohydrate metabolism (positive regulation of glucose import), fat metabolism (unsaturated fatty acid metabolic process, monocarboxylic acid binding), hormone and steroid metabolism (steroid metabolic process and hormone metabolic process, regulation of corticosteroid hormone secretion), and body growth (IGF ternary complex processes, negative regulation of tumor necrosis factor production, xenobiotic process, retinoid binding) were identified. Finally, when we compared the young bulls from dairy versus dual breed, the significant upregulated metabolic pathways for postnatal body growth (platelet-derived growth factor receptor binding, iron ion import into cell, and protein activation cascade) and transporting molecules (proton symporter activity) were identified; (iv) the significant downregulated metabolic pathways for fat metabolism (cholesterol biosynthetic process, cholesterol efflux, and ATP biosynthetic process), body growth and development (isoprenoid metabolic process, retinoic acid binding, negative regulation of protein processing), and energy metabolism (oxidoreductase activity) were identified.

In young growing bulls aged between 6 to 12 months, the liver is not fully metabolic stressed. However, it has major impact on feeding efficiencies [61,66,67,68], postnatal muscle growth and development [25,62,69,70], puberty [65], and carcass trait [24]. In cattle, several RNA-seq studies on liver and muscle tissues [13,61,66,67,68,69,70] have been reported and identified the metabolic pathways: (i) by comparing the liver transcriptome of Charolais, Angus, and KC beef breed; oxidation of fatty acids pathways were identified in KC and angus breed [13], (ii) by comparing the high- and low-RFI groups of Holstein and Jersey cows, several metabolic pathways that affect or regulate FE, including steroid hormone biosynthesis, retinol metabolism, starch and sucrose metabolism, ether lipid metabolism, arachidonic acid metabolism, and drug metabolism by cytochrome P450 were identified [61], (iii) by comparing the mild negative energy balance and severe negative energy balance in the liver of lactating HF cows with a severe negative energy balance, the steroid hormone biosynthesis pathway was identified [66], (iv) by comparing the different diets (low-impact, nutraceutical versus conventional diets) in young Belgian Blue × Holstein bulls, the metabolic pathways of cholesterol biosynthesis, liver X receptor/retinoid X receptors (LXR/RXR) activation, and glutathione metabolism were identified [67], (v) by comparing the rumen epithelial transcriptome of Hereford x Angus steers, energy generating pathways such as *glycolysis*, tricarboxylic acid cycle, and oxidative phosphorylation were identified [68], (vi) by comparing the grass- and grain-fed Angus steers in bovine *latissimus dorsi* transcriptome, metabolic pathways related to beef quality and animal walfare were identified [69], (vii) by comparing the muscle tissue of Nellore cattle with divergent meat tenderness, the glycine metabolic pathways was identified [70].

## 5. Conclusions

This is the first study to report the breed-specific DE gene-transcripts of native Polish cattle breeds and the Hereford reference breed using NGS-based transcriptome analysis of liver tissue, and provide a global view of the complexity of the bovine liver transcriptome. Our results demonstrate that the RNA-seq approach can be very useful for identifying the large amount of DE gene-transcripts (DEGs markers) in selected breeds of livestock animals. These identified hepatic DEG markers can serve as useful genetic tools to develop the gene assays for trait-associated studies, which can be effectively utilized in GS programs to improve the genomic resources available for cattle, especially for beef breeds. In this study, we provide the first transcriptome evidence that demonstrates cattle breed differences in the global gene-transcripts expression of dairy versus dual purpose versus beef cattle breeds. Our results clearly highlighted numerous hepatic gene-transcripts (*n* = 154, 120) and hepatic genes (*n* = 54, 395), as well as associated metabolic pathways in bovine liver transcriptome that were specific to the native breeds of Poland and the Hereford. Identification of breed-specific associate pathways in hepatic tissues of young bulls can further explore and understand the molecular regulations of the key metabolic pathways necessary in bovine postnatal body growth and muscle development, feeding efficiencies (RFI), puberty and carcass trait of young growing bulls from three cattle breeds. Finally, our study might eventually contribute to improve the cattle genome annotation by providing the accumulated biological knowledge of the functional groups of the genes that were found to be assigned to functional pathways in the liver transcriptome of dairy versus dual versus beef cattle breeds.

## Figures and Tables

**Table 1:** Mean phenotypic values of carcass traits in young growing bulls (*n* = 18) from three cattle breeds.

Breeds	*n*	Cold Carcass Weight (kg)	Carcass Yield (kg)	Right Half Carcass (kg)	Valuable Cut Meat (kg)	Meat (kg)	Bones (kg)	Fat (kg)
Polish-HF	6	188.9 ± 5.3	53 ± 2.4	94.9 ± 1.6	59.4 ± 1.9	42.3 ± 2.6	15.4 ± 3.6	9.9 ± 3.8
Polish-Red	6	166.6 ± 6.4	56.5 ± 3.6	86 ± 2.6	54.5 ± 3.4	40.7 ± 4.1	15.4 ± 5.7	10.9 ± 6.0
Hereford	6	193.9 ± 8.9	59.6 ± 4.6	100.2 ± 2.9	62.9 ± 3.1	45.4 ± 3.9	17.1 ± 5.6	13.0 ± 5.8

HF: Holstein Friesian.

**Table 2:** Hepatic gene expression NCBI database of Polish-Red, Polish-HF and Hereford cattle breeds.

Animal ID	Gene Expression Omnibus Acc. No.	Breed	Web-Link
CP19	GSM3138303	Polish Red	https://www.ncbi.nlm.nih.gov/geo/query/acc.cgi?acc=GSM3138303
CP20	GSM3138304	Polish Red	https://www.ncbi.nlm.nih.gov/geo/query/acc.cgi?acc=GSM3138304
CP21	GSM3138305	Polish Red	https://www.ncbi.nlm.nih.gov/geo/query/acc.cgi?acc=GSM3138305
CP22	GSM3138306	Polish Red	https://www.ncbi.nlm.nih.gov/geo/query/acc.cgi?acc=GSM3138306
CP23	GSM3138307	Polish Red	https://www.ncbi.nlm.nih.gov/geo/query/acc.cgi?acc=GSM3138307
CP24	GSM3138308	Polish Red	https://www.ncbi.nlm.nih.gov/geo/query/acc.cgi?acc=GSM3138308
CP27	GSM3138309	Polish HF	https://www.ncbi.nlm.nih.gov/geo/query/acc.cgi?acc=GSM3138309
CP28	GSM3138310	Polish HF	https://www.ncbi.nlm.nih.gov/geo/query/acc.cgi?acc=GSM3138310
CP29	GSM3138311	Polish HF	https://www.ncbi.nlm.nih.gov/geo/query/acc.cgi?acc=GSM3138311
CP30	GSM3138312	Polish HF	https://www.ncbi.nlm.nih.gov/geo/query/acc.cgi?acc=GSM3138312
CP31	GSM3138313	Polish HF	https://www.ncbi.nlm.nih.gov/geo/query/acc.cgi?acc=GSM3138313
CP32	GSM3138314	Polish HF	https://www.ncbi.nlm.nih.gov/geo/query/acc.cgi?acc=GSM3138314
CP35	GSM3138315	Hereford	https://www.ncbi.nlm.nih.gov/geo/query/acc.cgi?acc=GSM3138315
CP36	GSM3138316	Hereford	https://www.ncbi.nlm.nih.gov/geo/query/acc.cgi?acc=GSM3138316
CP37	GSM3138317	Hereford	https://www.ncbi.nlm.nih.gov/geo/query/acc.cgi?acc=GSM3138317
CP38	GSM3138318	Hereford	https://www.ncbi.nlm.nih.gov/geo/query/acc.cgi?acc=GSM3138318
CP39	GSM3138319	Hereford	https://www.ncbi.nlm.nih.gov/geo/query/acc.cgi?acc=GSM3138319
CP40	GSM3138320	Hereford	https://www.ncbi.nlm.nih.gov/geo/query/acc.cgi?acc=GSM3138320

**Table 3:** List of selected DE gene-transcripts for the validation of RNA-seq experiment by qRT-PCR.

Gene Full Name	*Calpain 11*	*Calpain 2*	*Insulin Like Growth Factor Binding Protein 1*	*Insulin Like Growth Factor Binding Protein 2*	*Insulin Like Growth Factor I*	*Insulin Like Growth Factor Binding Protein, Acid Labile Subunit (IGFALS)*	*Somatotropin*	*Family with Sequence Similarity 13 Member A*
Gene symbol	CAPN11	CAPN2	IGFBP1	IGFBP2	IGF-I	IGFALS	GH	FAM13A
Forward primer	GAACCTTCAA	CCAACATTGA	TAGCGTAAAT	GTGCAAGAT	GAGTGCAGG	CAGGTAACA	AGCCATCTG	CCTTTCTATTT
	CTGTCAAGCG	CGAGATTGACA	TGGCAGGGAA	GTCTCTGAACG	AAACAAGAACT	AGCTGGCCTA	TTGTTTGCCC	GAGCAGTGCC
Reverse primer	TTGAGTCGGT	ATTGTCTGCA	ACACTGTGTT	TGCTCGTTGT	TTGGTAGGT	GTGGTCCAG	TATTAGGAAA	CTGAGTCCTCT
	TCTGGCTTAT	ACTCAAAGGC	CCCATGTTTG	AGAAGAGATGA	CTTCTGGTGTT	GTAGAGTTTCT	GGACAGTGGGAG	GAACTTTGG
Ensembl Gene ID	ENSBTAG000	ENSBTAG000	ENSBTAG00	ENSBTAG00	ENSBTAG00	ENSBTAG000	ENSBTAG00	ENSBTAG000
	21066	12778	46768	5596	11082	33299	17220	11187
Ensembl Transcript ID	ENSBTAT00	ENSBTAT00	ENSBTAT00	ENSBTAT000	ENSBTAT00	ENSBTAT000	ENSBTAT00	ENSBTAT00
	4690	47532	64194	7349	14713	47326	22885	14855
BTA chromosome	23	16	4	2	5	25	19	6
Gene Start (bp)	17,807,298	27,781,671	76,720,883	1.05E + 08	66,532,877	1,366,647	48,768,618	37,355,568
Gene End (bp)	17,820,479	27,840,009	76,725,301	1.05E + 08	66,604,734	1,368,479	48,772,014	37,457,493
Transcript Start (bp)	17,807,298	27,781,671	76,720,883	1.05E + 08	66,532,877	1,366,647	48,768,618	37,355,568
Transcript length	2675	3179	917	1141	862	1833	817	5125

## Supplementary Materials

The following are available online at https://www.mdpi.com/2306-7381/6/2/36/s1, All hepatic expression data of cattle breeds (*n* = 18) were submitted to NCBI acc. No.: GSE114233 https://www.ncbi.nlm.nih.gov/geo/query/acc.cgi?acc=GSE114233 (Table 2). Figure S1: Workflow of RNA-seq laboratory method; Figure S2: RIN values of investigated Polish-Red young bulls; Figure S3: RIN values of investigated Polish-HF young bulls; Figure S4: RIN values of investigated Hereford young bulls; Figure S5: Identification of GO/pathway terms specific for upregulated genes as shown in cluster-1 and cluster-2 for all DE genes expressed in liver tissues by comparing the Polish-Red versus Hereford cattle breeds using Cytoscape-ClueGo. (On Right: Functional terms in ClueGo chart. On left: Functional groups in ClueGo overview); Figure S6: Identification of GO/pathway terms specific for downregulated genes as shown in cluster-1 and cluster-2 for all DE genes expressed in liver tissues by comparing the Polish-Red versus Hereford cattle breeds using Cytoscape-ClueGo. (On Right: Functional terms in ClueGo chart. On left: Functional groups in ClueGo overview); Figure S7: Identification of GO/pathway terms specific for upregulated genes as shown in cluster-1 and cluster-2 for all DE genes expressed in liver tissues by comparing the Polish-HF versus Hereford cattle breeds using Cytoscape-ClueGo. (On Right: Functional terms in ClueGo chart. On left: Functional groups in ClueGo overview); Figure S8: Identification of GO/pathway terms specific for downregulated genes as shown in cluster-1 and cluster-2 for all DE genes expressed in liver tissues by comparing the Polish-HF versus Hereford cattle breeds using Cytoscape-ClueGo. (On Right: Functional terms in ClueGo chart. On left: Functional groups in ClueGo overview); Figure S9: Identification of GO/pathway terms specific for upregulated genes as shown in cluster-1 and cluster-2 for all DE genes expressed in liver tissues by comparing the Polish-HF versus Polish-Red cattle breeds using Cytoscape-ClueGo. (On Right: Functional terms in ClueGo chart. On left: Functional groups in ClueGo overview); Figure S10: Identification of GO/pathway terms specific for downregulated genes as shown in cluster-1 and cluster-2 for all DE genes expressed in liver tissues by comparing the Polish-HF versus Polish-Red cattle breeds using Cytoscape-ClueGo. (On Right: Functional terms in ClueGo chart. On left: Functional groups in ClueGo overview).

Table S1: Identification of all hepatic genes without cut-off *p* value and with cutoff *p* value (*p* < 0.01), expressed in bovine liver tissues by comparing the Polish-Red vs. Hereford cattle breeds using DEseq and EdgeR pipelines; Table S2: Identification of all hepatic genes without cut-off *p* value and with cutoff *p* value (*p* < 0.01), expressed in bovine liver tissues by comparing the Polish-HF vs. Hereford cattle breeds using DEseq and EdgeR pipelines; Table S3: Identification of all hepatic genes without cut-off *p* value and with cutoff p value (*p* < 0.01), expressed in bovine liver tissues by comparing the Polish-HF vs. Polish-Red cattle breeds using DEseq and EdgeR pipelines; Table S4: Identifications of all the hepatic non-DE genes counts (without cut-off p values) expressed in bovine liver tissues by comparing all three breeds using DEseq and EdgeR pipelines as shown in Venn Figure 1; Table S5: Identifications of significant DE genes counts (*p* < 0.01) expressed in bovine liver tissues by comparing all three breeds using DEseq and EdgeR pipelines as shown in Venn Figure 2; Table S6: Identification of top ranked 150 GO terms (GO-MF, GO-BP, and GO-CC) in bovine liver tissues using DEseq and EdgeR pipelines (Sheet 1 to 3); Table S7: Analysis of functional distribution of DE genes in liver tissues by comparing the Polish-Red vs. Hereford cattle breeds using Cytoscape-ClueGo; Table S8: Analysis of functional distribution of DE genes in liver tissues by comparing the Polish-HF vs. Hereford cattle breeds using Cytoscape-ClueGo; Table S9: Analysis of functional distribution of DE genes in liver tissues by comparing the Polish-HF vs. Polish-Red cattle breeds using Cytoscape-ClueGo; Table S10: The gene network and biological processes for genes specific to all DE genes in liver tissues by comparing the Polish-Red vs. Hereford cattle breeds using Cytoscape-ClueGo. The distribution of two clusters visualized on the functionally grouped network with terms as nodes linked based on their kappa score level (≥0.3), where only the label of the most significant term per group is shown. The node size represents the term enrichment significance. Terms with up/downregulated genes are shown in red/blue, respectively. The colour gradient shows the gene proportion of each cluster associated with the term; Table S11: The gene network and biological processes for genes specific to all DE genes in liver tissues by comparing the Polish-HF vs. Hereford cattle breeds using Cytoscape-ClueGo. The distribution of two clusters visualized on the functionally grouped network with terms as nodes linked based on their kappa score level (≥0.3), where only the label of the most significant term per group is shown. The node size represents the term enrichment significance. Terms with up/downregulated genes are shown in red/blue, respectively. The colour gradient shows the gene proportion of each cluster associated with the term; Table S12: The gene network and biological processes for genes specific to all DE genes in liver tissues by comparing the Polish-HF vs. Polish-Red cattle breeds using Cytoscape-ClueGo. The distribution of two clusters visualized on the functionally grouped network with terms as nodes linked based on their kappa score level (≥0.3), where only the label of the most significant term per group is shown. The node size represents the term enrichment significance. Terms with up/downregulated genes are shown in red/blue, respectively. The colour gradient shows the gene proportion of each cluster associated with the term.
