# Supplementary material for: Comparative Analysis of the Liver Transcriptome among Cattle Breeds Using RNA-seq
Source: Vet Sci. 2019 Mar 29;6(2):36. doi: 10.3390/vetsci6020036 (PMC6631511; doi:10.3390/vetsci6020036)
Supplement: Supplementary file 1 [file vetsci-06-00036-s001.zip › Supp Figures-S1 to S10.docx]

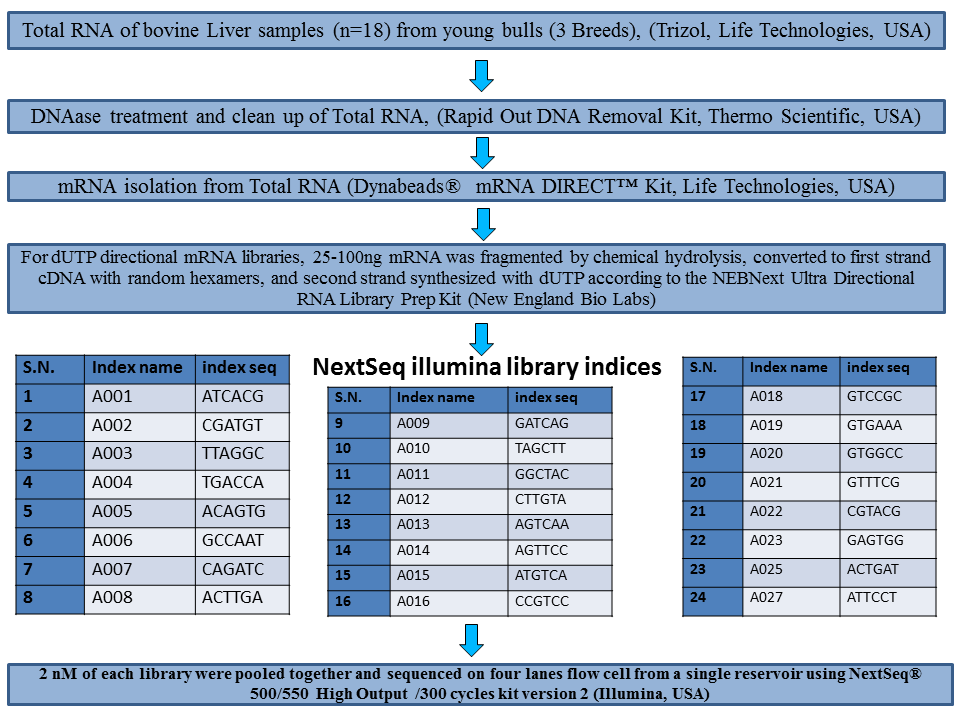


**Figure S1.** Workflow of RNA-seq laboratory method.


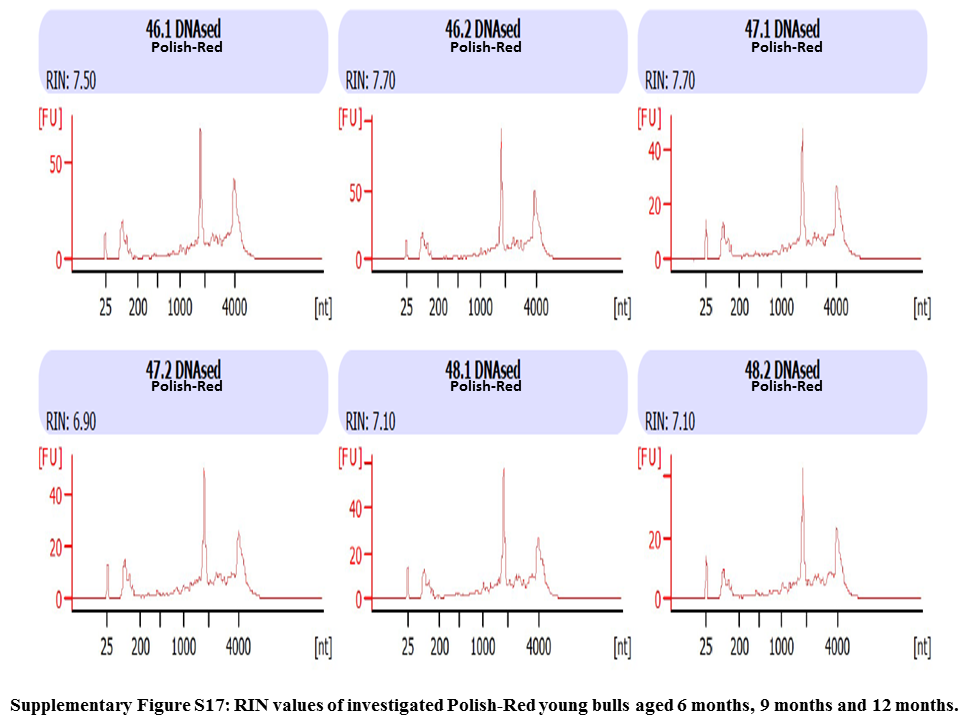


**Figure S2.** RIN values of investigated Polish-Red young bulls aged 6 month, 9 month and 12 month.


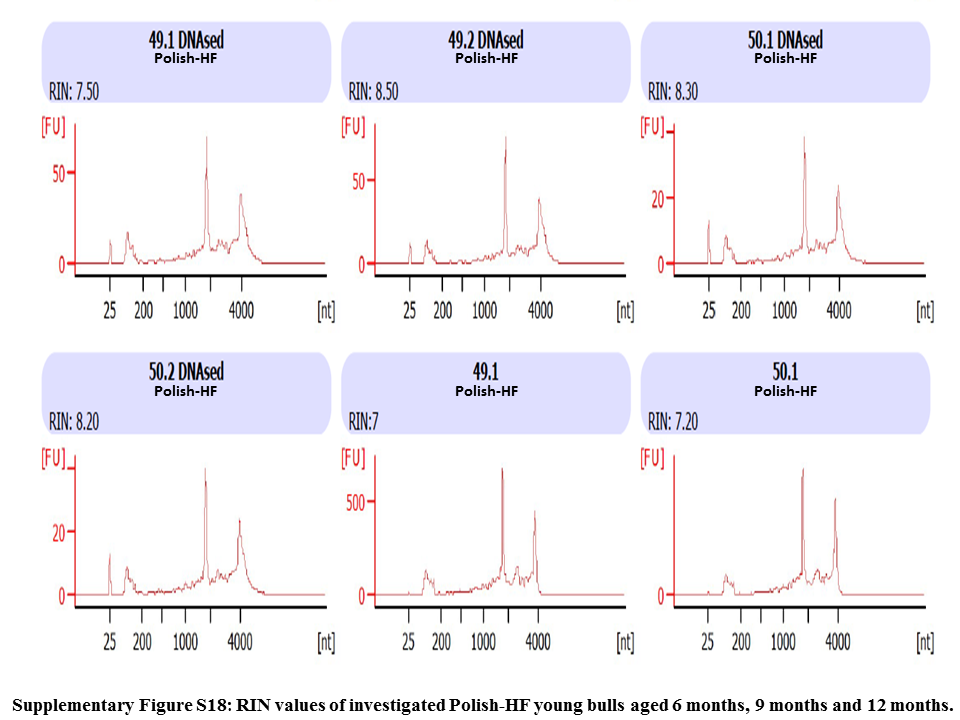


**Figure S3.** RIN values of investigated Polish-HF young bulls aged 6 month, 9 month and 12 month.


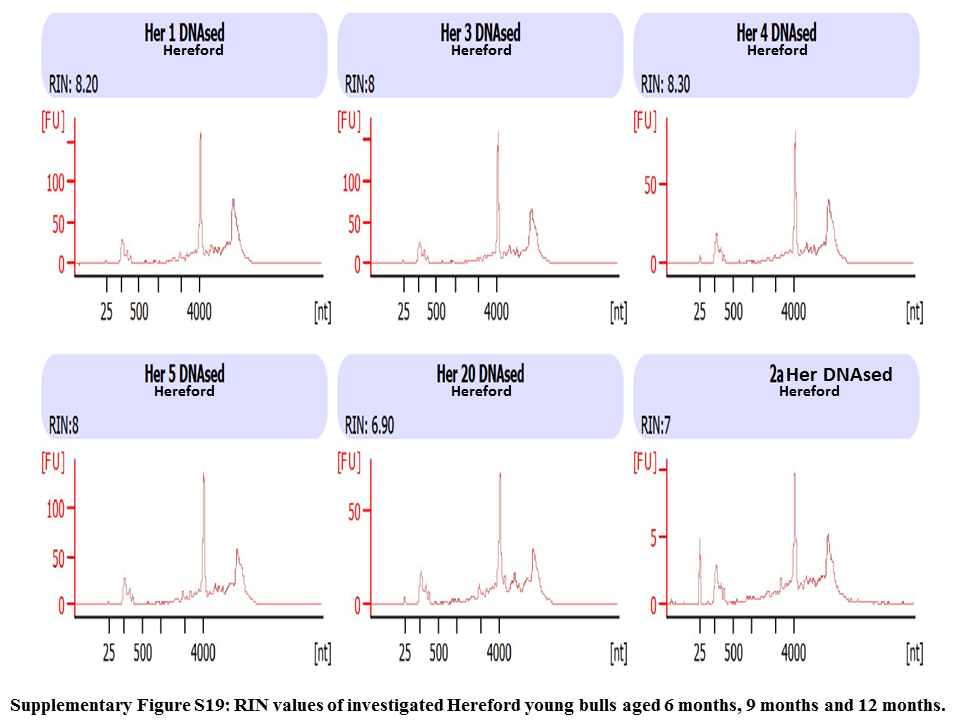


**Figure S4.** RIN values of investigated Hereford young bulls aged 6 month, 9 month and 12 month.

**Figure S5.** Identification of GO/pathway terms specific for upregulated genes as shown in cluster-1 and cluster-2 for all DE gene-transcripts expressed in liver tissues by comparing the Polish-Red vs. Hereford cattle breeds using Cytoscape-ClueGo. On Right: Functional terms in ClueGo chart, On left: Functional groups in ClueGo overview.

.

**Figure S6.** Identification of GO/pathway terms specific for downregulated genes as shown in cluster-1 and cluster-2 for all DE gene-transcripts expressed in liver tissues by comparing the Polish-Red vs. Hereford cattle breeds using Cytoscape-ClueGo. On Right: Functional terms in ClueGo chart, On left: Functional groups in ClueGo overview.

**Figure S7.** Identification of GO/pathway terms specific for upregulated genes as shown in cluster-1 and cluster-2 for all DE gene-transcripts expressed in liver tissues by comparing the Polish-HF vs. Hereford cattle breeds using Cytoscape-ClueGo. On Right: Functional terms in ClueGo chart, On left: Functional groups in ClueGo overview.

**Figure S8.** Identification of GO/pathway terms specific for downregulated genes as shown in cluster-1 and cluster-2 for all DE gene-transcripts expressed in liver tissues by comparing the Polish-HF vs. Hereford cattle breeds using Cytoscape-ClueGo. On Right: Functional terms in ClueGo chart, On left: Functional groups in ClueGo overview.

**Figure S9.** Identification of GO/pathway terms specific for upregulated genes as shown in cluster-1 and cluster-2 for all DE gene-transcripts expressed in liver tissues by comparing the Polish-HF vs. Polish-Red cattle breeds using Cytoscape-ClueGo. On Right: Functional terms in ClueGo chart, On left: Functional groups in ClueGo overview.

**Figure S10.** Identification of GO/pathway terms specific for downregulated genes as shown in cluster-1 and cluster-2 for all DE gene-transcripts expressed in liver tissues by comparing the Polish-HF vs. Polish-Red cattle breeds using Cytoscape-ClueGo. On Right: Functional terms in ClueGo chart, On left: Functional groups in ClueGo overview.
